# Application of HPLC-QQQ-MS/MS and New RP-HPLC-DAD System Utilizing the Chaotropic Effect for Determination of Nicotine and Its Major Metabolites Cotinine, and *trans*-3′-Hydroxycotinine in Human Plasma Samples

**DOI:** 10.3390/molecules27030682

**Published:** 2022-01-20

**Authors:** Jacek Baj, Wojciech Flieger, Dominika Przygodzka, Grzegorz Buszewicz, Grzegorz Teresiński, Magdalena Pizoń, Ryszard Maciejewski, Jolanta Flieger

**Affiliations:** 1 Department of Anatomy, Medical University of Lublin, Jaczewskiego 4, 20-090 Lublin, Poland; ryszard.maciejewski@umlub.pl; 2 Department of Forensic Medicine, Medical University of Lublin, 20-090 Lublin, Poland; dominikaprzygodzka@umlub.pl (D.P.); g.buszewicz@umlub.pl (G.B.); grzegorz.teresinski@umlub.pl (G.T.); 3 Department of Analytical Chemistry, Medical University of Lublin, Chodźki 4A, 20-093 Lublin, Poland; magdalena.pizon@umlub.pl

**Keywords:** nicotine, cotinine, *trans*-3′-hydroxycotinine, RP-HPLC-DAD, HPLC-QQQ-MS/MS chaotropic effect

## Abstract

The routine techniques currently applied for the determination of nicotine and its major metabolites, cotinine, and *trans*-3′-hydroxycotinine, in biological fluids, include spectrophotometric, immunoassays, and chromatographic techniques. The aim of this study was to develop, and compare two new chromatographic methods high-performance liquid chromatography coupled to triple quadrupole mass spectrometry (HPLC-QQQ-MS/MS), and RP-HPLC enriched with chaotropic additives, which would allow reliable confirmation of tobacco smoke exposure in toxicological and epidemiological studies. The concentrations of analytes were determined in human plasma as the sample matrix. The methods were compared in terms of the linearity, accuracy, repeatability, detection and quantification limits (LOD and LOQ), and recovery. The obtained validation parameters met the ICH requirements for both proposed procedures. However, the limits of detection (LOD) were much better for HPLC-QQQ-MS/MS (0.07 ng mL^−1^ for *trans*-3′-hydroxcotinine; 0.02 ng mL^−1^ for cotinine; 0.04 ng mL^−1^ for nicotine) in comparison to the RP-HPLC-DAD enriched with chaotropic additives (1.47 ng mL^−1^ for *trans*-3′-hydroxcotinine; 1.59 ng mL^−1^ for cotinine; 1.50 ng mL^−1^ for nicotine). The extraction efficiency (%) was concentration-dependent and ranged between 96.66% and 99.39% for RP-HPLC-DAD and 76.8% to 96.4% for HPLC-QQQ-MS/MS. The usefulness of the elaborated analytical methods was checked on the example of the analysis of a blood sample taken from a tobacco smoker. The nicotine, cotinine, and *trans*-3′-hydroxycotinine contents in the smoker’s plasma quantified by the RP-HPLC-DAD method differed from the values measured by the HPLC-QQQ-MS/MS. However, the relative errors of measurements were smaller than 10% (6.80%, 6.72%, 2.04% respectively).

## 1. Introduction

Nicotine is a pyridine chiral alkaloid, most abundant in tobacco leaves (*Nicotiana tabacum* L.), and in smaller amounts in tomatoes and other plants in the Solanaceae family. Nicotine is known to be a highly addictive neurotoxin [1]. The lethal dose (LD_50_) value for nicotine is approx. 1–1.5 mg per kg of body weight. The median LD_50_ for nicotine is assumed to be 0.8 mg/kg for adults [2]. The Royal Children’s Hospital Melbourne in the Clinical Practice Guidelines reports the potentially lethal dose of nicotine for children is greater than 0.5 mg/kg [3].

The main source of nicotine is tobacco smoke which contains moreover about 5300 chemicals such as carbon monoxide, benzene, hydrogen cyanide, tar, formaldehyde, etc. [4]. For this reason, tobacco smoke is listed as the main cause of cancer in many organs, including the lungs, nose, mouth, pharynx, esophagus, larynx [5]. Tobacco-attributable diseases also include heart diseases, chronic respiratory diseases, and diabetes—all of which may increase the severity of COVID-19 infection. The 2019 WHO report states that almost a third of adults worldwide are regularly exposed to tobacco smoke [6]. In the last years, smoking prevalence among people aged over 15 years has fallen from 22.7% to 17.5%, however, tobacco is still responsible for one of the causes of more than 8 million premature deaths in the world [7].

Nicotine is perfectly absorbed from cigarette smoke in the alveoli and through the epithelium lining the mouth [8,9,10,11]. After getting into the bloodstream, it transforms into an ionized form in about 70% due to the blood pH of 7.4. Only 5% of nicotine is bound to plasma proteins. The main metabolite of nicotine is cotinine, which is formed as a result of the transformation of about 80% of inhaled nicotine [10] by the highly genetically variable enzyme CYP2A6 [12]. Due to its long half-life (16–20 h), cotinine is often determined in blood, urine, and saliva as a biomarker of exposure to tobacco smoke [13,14]. The main metabolite of cotinine is 3′-hydroxycotinin, detected in the urine and plasma of smokers. However, several other metabolites of cotinine have also been identified in the human body, such as 5-hydroxycotinin, N-cotinine oxide, cotinine methonium ion, glucuronide cotinines, and norcotinine [10,15]. Biomarkers of exposure to tobacco smoke are also thiocyanates measured in the saliva of smokers [16].

Various techniques are used to determine the level of nicotine metabolites in body fluids, such as immunoassays e.g., radioimmunoassay (RIA), enzyme-linked immunosorbent assay (ELISA), fluorescence immunoassay (FIA), spectrophotometric, and chromatographic techniques [13,17,18,19,20,21]. The most common methods recognized for the quantification of nicotine and its metabolites are gas (GC) and liquid chromatography (LC) coupled with flame-ionization detection (FID), ultraviolet (UV), and mass spectrometry (MS) detectors. Makoto Yasuda reported an HPLC with fluorometric detection (FD) method for nicotine, cotinine, nicotinic acid, and nicotinamide determination [22]. The authors used the fluorescence of nicotine and cotinine, whereas the remaining two metabolites’ fluorescence was enhanced with derivatization using hydrogen peroxide. However, in the described procedure of sample preparation, analytes were extracted from alkalinized human serum via liquid–liquid extraction using hazardous chloroform.

Currently, chromatographic techniques are combined with mass spectrometry, for example liquid chromatography-mass spectrometry (LC-MS) [23,24,25,26,27], liquid chromatography-tandem mass spectrometry (LC-MS/MS) [28,29,30], and gas chromatography-mass spectrometry (GC–MS) [31,32].

So far, the determination of nicotine using LC-MS/MS has been described in various biological matrices, e.g., urine, [33,34,35,36,37,38,39,40,41,42,43,44], plasma and serum [25,45,46,47,48], saliva [37,42,47], and hair [49,50,51,52,53]. The LOD values reported for nicotine are most often in the range 1.00–10.0 ng mL^−1^. The cotinine was detected on the level of 2 ng mL^−1^ [25] or, even below 0.1 ng mL^−1^ according to Jacob et al. [26]. An ultra-high-performance liquid chromatography UHPLC/HPLC mixed in tandem with a triple quadrupole system enabled the measurement of serum nicotine with a LOD value of approximately 0.050 ng mL^−1^ [54,55]. In this case, the chromatographic separation was conducted on a reversed-phase column eluted by a linear gradient of mobile phase composed of 0.05% ammonium hydroxide and acetonitrile. To separate nicotine and its major metabolites, cotinine, *trans*-3′-hydroxycotinine, nicotine N-oxide and cotinine N-oxide, the hydrophilic interaction chromatography-tandem mass spectrometry (HILIC-MS/MS) was described by Marclay and Saugy [41]. The authors applied the stationary phase of Luna HILIC 200 cross-linked diol with gradient elution mode from 98% to 35% acetonitrile in 10 mmol L^−1^ of ammonium formate buffer at pH 3.0.

The current study is the first attempt to determine nicotine and its two major metabolites cotinine, and *trans*-3′-hydroxycotinine by the reverse-phase HPLC system, enriched with chaotropic additives. As predicted previously by Flieger [56,57,58], the addition of the salts with chaotropic properties has a favorable effect on retention and ensured improved selectivity and efficiency of the reversed-phase chromatographic system towards basic analytes. Nicotine alkaloids as compounds possessing basic functional groups able to ionize require special chromatographic conditions. When they are analyzed by the use of strong acidic mobile phases, unfavorable silanol effects usually provide increase in retention together with the loss of column efficiency. 

The aim of the current study is to investigate the usefulness of the classic RP-HPLC-DAD system enriched with the addition of a chaotropic salt for the quantitative determination of nicotine, cotinine, and *trans*-3′-hydroxycotinine in human plasma samples. The method was validated and compared to HPLC-QQQ-MS/MS as a reference. Both methods were compared in terms of the linearity, accuracy, repeatability, recovery as well as detection and quantification limits. Finally, the results of determination of examined analytes in real plasma samples of tobacco smokers were compared and statistically evaluated.

## 2. Results

### 2.1. HPLC-DAD of trans-3′-Hydroxycotinine, Cotinine, Nicotine

**Figure 1:**
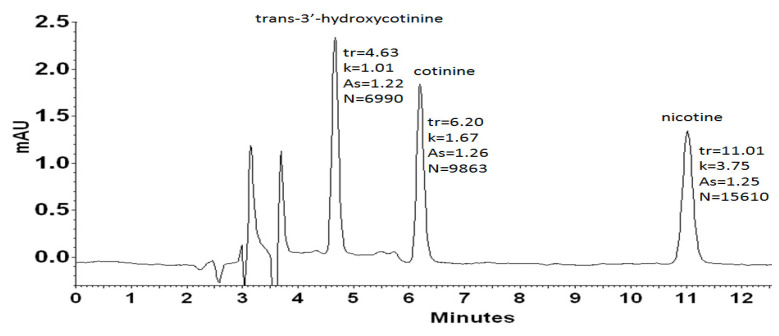
HPLC-DAD chromatogram obtained for solution of standards: *trans*-3′-hydroxycotinine (tr = 4.63 min), cotinine (tr = 6.16 min), nicotine (tr = 11.01 min) at the concentration level of 500 ng L^−1^. The analysis was performed using an Agilent 5 HC-C18(2) (250 × 4.6 mm I.D.) column. The mobile phase was acetonitrile (10%, *v*/*v*), 20 mM phosphate buffer pH = 2.7 containing 30 mM NaPF_6_ in the whole mobile phase. The DAD detection was set at 260 nm.

All analyzed compounds, i.e., nicotine, cotinine, and *trans*-3′-hydroxycotinine, contain nitrogen, which may be in protonated or unprotonated form, depending on the pH of the reaction medium. Nicotine containing a pyridine ring (p*K*_a_ 2.96) and a pyrrolidine ring (p*K*_a_ 8.07) [59,60] occurs as a diprotonated cation with a charge on both of the ionizable moieties in an acidic aqueous medium. The p*K*_a_ value for both cotinine and *trans*-3′-hydroxycotinine is about 4.5 also due to the protonation of nitrogen on the pyridine ring. Thus, the above analytes exist as the cationic forms in the acidic eluent system (pH 2.7). The analytes ionization is a source of strong electrostatic interactions with the counter-anion of the chaotropic salt providing the retention of analytes with distinct hydrophilic properties (log P of nicotine is 0.93, log P of cotinine is 0.07, and log P of *trans*-3′-hydroxycotinin equals −1.45) on a hydrophobic RP sorbent. The use of the mobile phase with pH smaller than 3, has also other advantages in the case of UV detection mode because the protonated forms possess higher absorbance at 260 nm in comparison to the ones existing at higher pH. Figure 1 presents a chromatogram of a mixture containing *trans*-3′-hydroxycotinine, cotinine, and nicotine obtained by the use of RP-HPLC-DAD system utilizing sodium hexafluorophosphate as a chaotropic additive to the acidic mobile phase.

**Figure 2:**
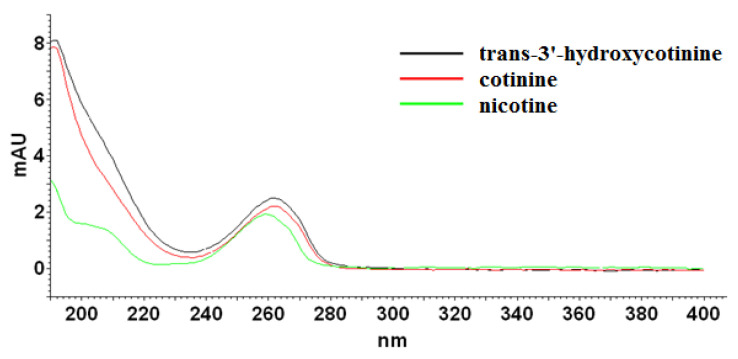
UV-absorption spectra of nicotine, cotinine, and *trans*-3′-hydroxycotinine standards measured from 220 to 400 nm.

As it can be seen, the applied eluent system permitted complete resolution of the analyzed compounds in an analysis time lower than 15 min. The chaotropic additive ensured satisfactory efficiency expressed in the theoretical plates number (N) in the range of 27,960 to 62,440, and peak symmetry (As) no more than 1.5 at each case. According to spectra collected for examined compounds (Figure 2) in the range from 220 nm to 400 nm, the detection wavelength was established at 260 nm. Further optimization experiments were performed at the chosen analytical wavelength.

**Figure 3:**
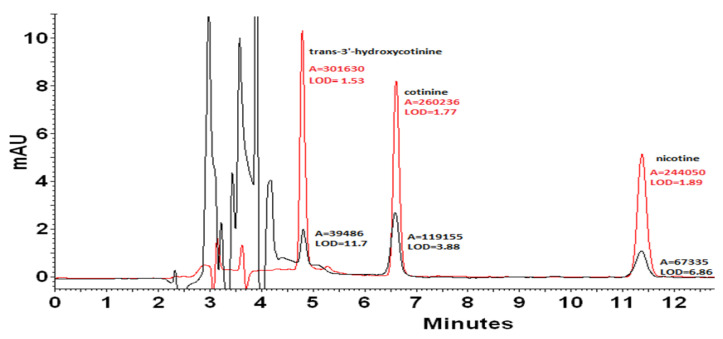
Chromatogram of a mixture of standards with a concentration of 2 mg mL^−1^ dissolved in methanol (black line) and in the mobile phase (red line). The detection limits [ng mL^−1^] were estimated considering the analyte concentration that produces a chromatographic peak having a height equal to three times the standard deviation of the baseline noise.

In the case of quantitative analysis, a very important aspect is to achieve the low limits of detection (LOD). A significant reduction in the LOD value can be obtained thanks to the preparation of samples in the mobile phase (Figure 3). As a result, it was possible to reduce the LOD value for nicotine 3.6 times from 6.86 to LOD = 1.89 ng mL^−1^. The LOD value for cotinine decreased from 3.88 to 1.77 ng mL^−1^. The best result was obtained for *trans*-3′-hydroxycotinine, for which the LOD value was reduced 7.6 times from 11.7 to 1.53 ng mL^−1^. As can be seen, chaotropic additive provided a higher signal level as well as significantly reduced unwanted noise. Therefore, samples for further quantification were prepared by dilution with the mobile phase.

#### 2.1.1. HPLC-DAD Method Validation 

The matrix effect usually comes from endogenous components coming from the sample or contaminants introduced in the pretreatment process. The matrix effect can be evaluated as the ratio between the slopes of the linear calibration equations of analyte dissolved in the blank matrix per that one obtained for analyte dissolved in the organic solvent. In DAD detection mode, the investigated analytes cotinine, nicotine, and *trans*-3′-hydroxycotinine demonstrated a relatively strong matrix effect equaling 86.58%, 94.55%, and 83.42% respectively. In the aim to avoid the influence of the matrix on analytes quantification, the standard addition method (fortification method) ensuring the stability and accuracy of the results was applied in subsequent experiments.

Linearity, LOD and LOQ values were determined for all analytes using an artificial plasma matrix that was spiked with the analytes at seven different concentration levels. The obtained mixtures were further analyzed by the extraction procedure described in the Section 4.2.2. The calibration curves were linear in the range from 10 to 1200 ng mL^−1^ with the determination coefficients (R^2^) being greater than 0.9980. The least-squares linear regression analysis was used to determine the statistic parameters which were collected in Table 1. 

**Figure 4:**
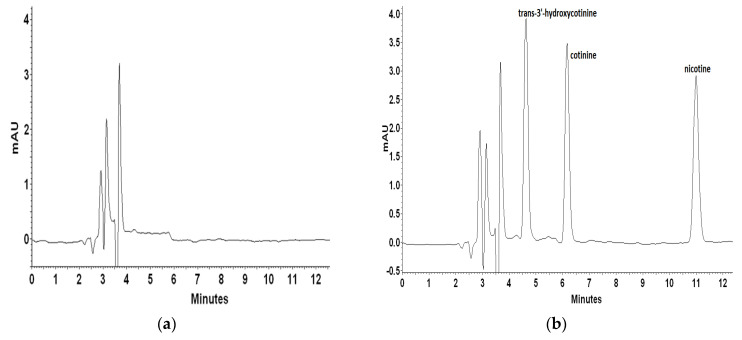

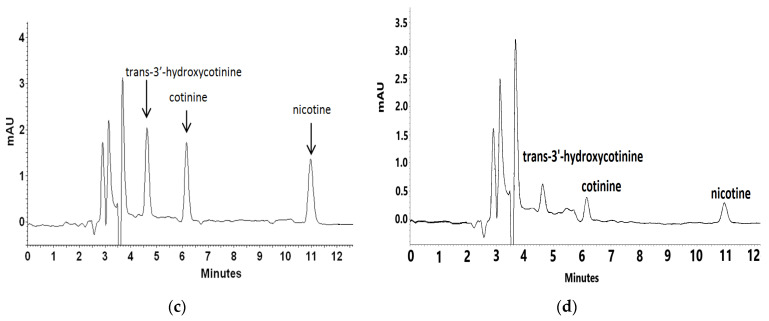
Representative chromatograms of blank plasma sample (**a**) and plasma spiked with trans-3′-hydroxycotinine, cotinine, nicotine standards at a concentration of 1000 ng L^−1^ (**b**), 500 ng L^−1^ (**c**), and 100 ng L^−1^ (**d**).

For recovery and repeatability examination blank plasma matrix was spiked with mixed standard solutions on three concentration levels. Each level was quantified six times to calculate the recovery and relative standard deviation (RSD). Inter- and intra-day precision and accuracy data for nicotine and its metabolites were determined with the LQC (100 ng mL^−1^), MQC (500 ng mL^−1^), and HQC (1000 ng mL^−1^) samples. Figure 4 presents a representative chromatogram of blank artificial plasma sample and plasma spiked with *trans*-3′-hydroxycotinine, cotinine, and nicotine on different concentration levels. 

The analytes concentration was calculated from the calibration curve. Recovery (%) was calculated by comparing the measured analyte concentration to the expected value. Intra-day data were assessed by comparing data from within one run (n = 3 for each QC). Inter-day validation data were obtained from analyses conducted on three subsequent days (n = 3). Accuracy was in the range of 96.66% to 99.39%. Coefficients of variation (CVs) were <5% for intraday precision and <6% for interday precision. Obtained results summarized in Table 2 were considered acceptable for all quality samples considering criteria established by the US FDA, that is 85–115% for accuracy, and ±15% for precision [61,62].

#### 2.1.2. Analysis of Real Plasma Sample from a Tobacco-Smoking Patient

The standard additions calibration method was used to determine the concentration of the investigated analytes in a real human plasma sample coming from the tobacco smoker. This method is commonly found when working with clinical, biological, or food samples mainly to avoid the matrix effect and unexpected interferences from matrix components. In a smoker’s plasma sample, nicotine and its metabolites were expected at rather low concentration levels. To avoid loss of precision as a result of sample dilution, the supernatant obtained after mixing plasma with an organic solvent was lyophilized and dissolved in a small volume of 300 µL of the mobile phase. Obtained results were collected in Table 3.

### 2.2. HPLC-QQQ-MS/MS of trans-3′-Hydroxycotinine, Cotinine, Nicotine

**Figure 5:**
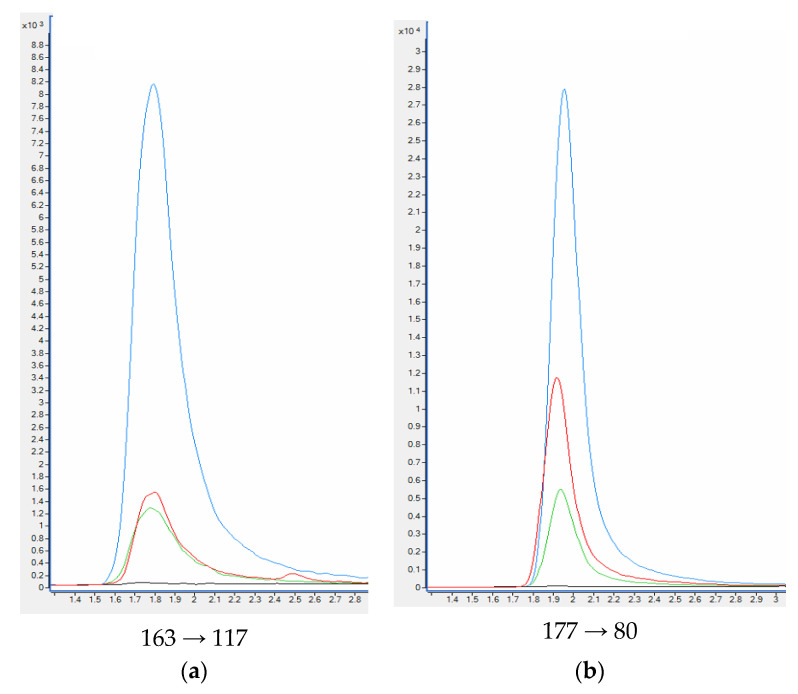

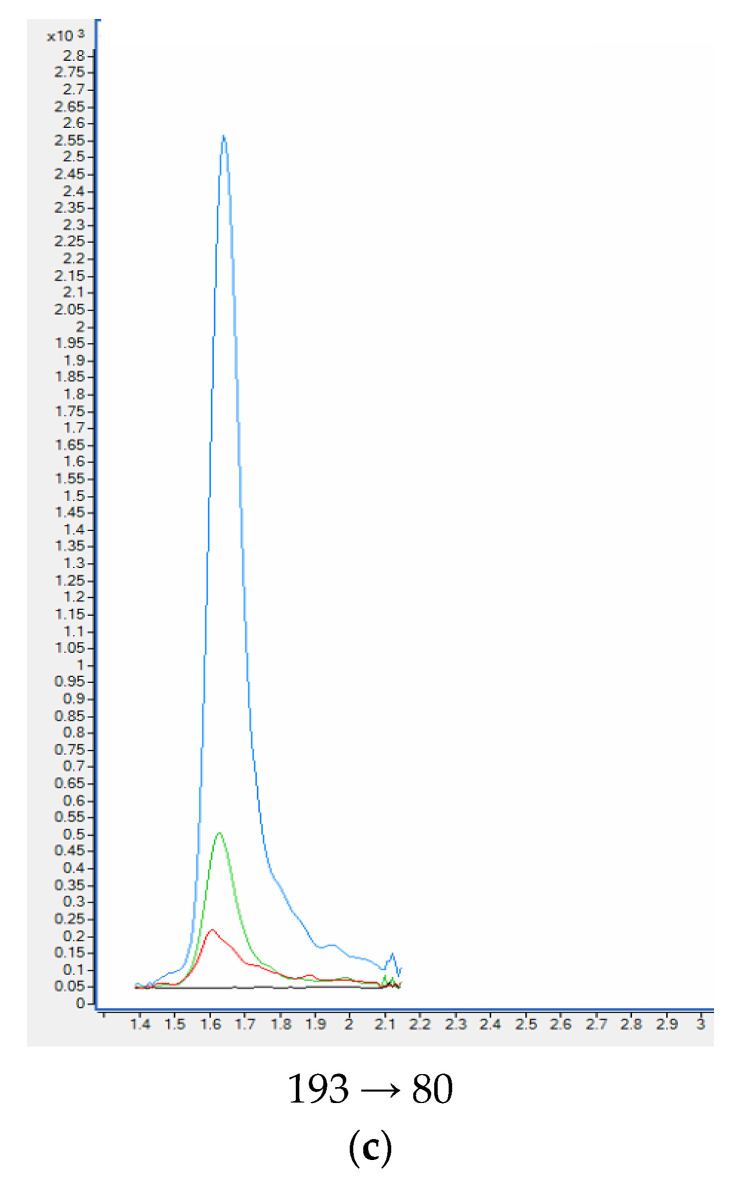
HPLC-QQQ-MS/MS chromatograms of quantitative transition of nicotine (**a**) and metabolites: cotinine (**b**), *trans*-3′-hydroksycotinine (**c**) from the blank plasma sample, plasma samples used in the validation process and smoker’ plasma sample. Plasma sample spiked with 100 ng mL^−1^ of analytes (blue); Plasma sample spiked with 20 ng mL^−1^ of analytes (green); smoker’ plasma sample (red); blank plasma sample (black).

**Figure 6:**
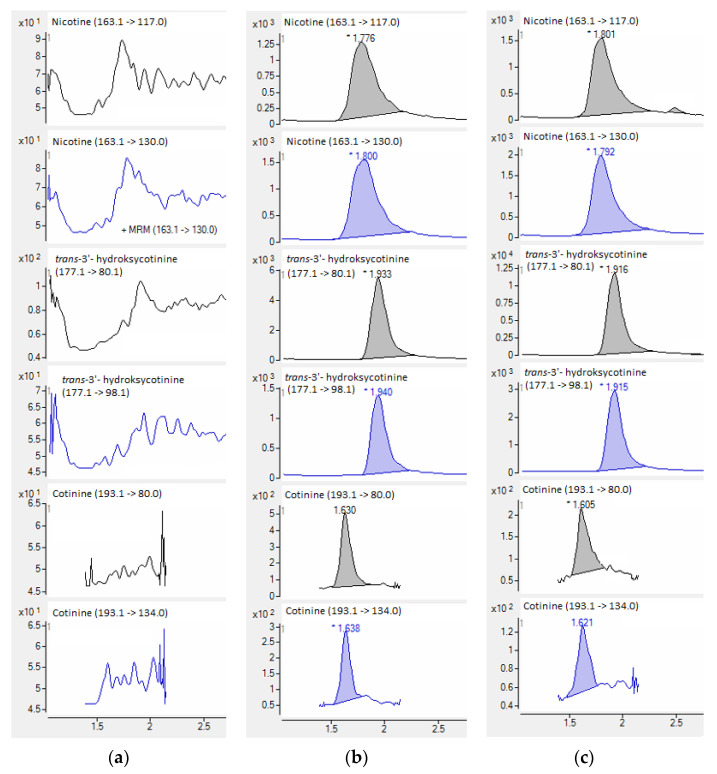
HPLC-QQQ-MS/MS chromatograms of quantitative (black) and confirmatory (blue) transitions of nicotine and metabolites from the blank plasma sample (**a**), plasma samples used in the validation process (**b**), and smoker’ plasma sample (**c**).

When standards and deuterated internal standards were added to the blank plasma sample, all analytes showed clear well-defined peaks without any interferences. The intensities of the peaks were above 200-times higher in comparison to the background signals detected for the blank plasma samples (Figure 5). HPLC-QQQ-MS/MS chromatograms of nicotine and examined metabolites are collected in Figure 6.

#### Validation of HPLC-QQQ-MS/MS

Nicotine and its metabolites calibration curves were prepared in the range 2.0 to 200 ng mL^−1^. Calibration curve parameters were collected in Table 4. For each of the compounds tested, the curves had an R^2^ above 0.999 (only for nicotine it was 0.9989). Smoker’s plasma samples were analyzed together with two QC samples (20 and 200 ng mL^−1^).

The recovery study was performed for each analyte at two concentration levels of 20 and 200 ng mL^−1^. As can be seen in Table 5, the recovery of analytes was concentration-dependent. For the smaller concentration of 20 ng mL^−1^ the percentage of recovery was in the range from 76.8% to 81.9%, whereas for ten times higher concentration levels, the recovery was between 93.1 and 96.4%. In a typical smoker’s plasma sample, the investigated analytes were detected on the level of 7.544, 50.180, 19.588 ng mL^−1^ for *trans*-3′-hydroxy-cotinine, cotinine, nicotine respectively by HPLC-QQQ-MS/MS.

### 2.3. Comparison of the Results Obtained by RP-HPLC-DAD with HPLC-QQQ-MS/MS

The nicotine, cotinine, and *trans*-3′-hydroxycotinine contents in the smoker’s plasma quantified by the RP-HPLC-DAD method differ from the values measured by the HPLC-QQQ-MS/MS. The relative error values were 6.80%, 6.72%, 2.04%, respectively. It should be noted, however, that the marked quantities are of the same order of magnitude. To statistically compare the series of measurements, linear regression analysis or a Bland-Altman plot (B & A is known as the Tukey mean difference plot) could be used. It could be possible in the case of much more samples from smokers. Unfortunately, blood collection is an invasive procedure, so in the future, the applicability of the method should be checked on other biological matrices, such as urine or saliva. The results were compared utilizing a statistical significance *t*-test. The results obtained for analysis of *trans*-3′-hydroxycotinine, cotinine and nicotine in plasma samples using HPLC-DAD and *HPLC-QQQ-MS/MS* showed that there are no significant differences between these two chromatographic techniques using the *t*-test at a significance level of 0.05 on a two-tailed test for 2 degrees of freedom.

## 3. Discussion

The presence of nicotine metabolites in body fluids can be confirmed by various techniques, differing in implementation costs, time consumption, selectivity, reliability and efficiency. Despite the availability of a wide range of analytical tools, the most technologically advanced ones are not standard laboratory equipment, and their operation is too expensive to be used for epidemiological, toxicological and even clinical screening, which prefer quick and cheap analytical techniques.

Our study concerns a new method for the determination of nicotine, cotinine and *trans*-3′-hydroxycotinin in the blood plasma, using the classic RP-HPLC-DAD system modified with the addition of NaPF_6_ chaotropic salt at millimolar concentration. The obtained results regarding the selectivity of the separation exceed the previous HPLC separations, which have been described in the literature so far. The addition of a chaotropic salt had a positive effect on the achieved detection and quantification limits. It is known, however, that HPLC-DAD cannot compete with tandem mass spectrometry, which allows for unique identification of compounds and provides the best possible sensitivity of the method. It should be emphasized, that the obtained limit of quantification (LLOQ) for cotinine was even smaller than 10 ng mL^−1^. This value is sufficient for selecting smokers, because according to Kim [63] the serum cotinine cutoff value range of 10–20 ng/mL, serving as a cut-off point to verify patients according to their tobacco status. 

Our study confirmed the advantage of chaotropic additives in RP systems over gradient analysis or multi-component eluents in order to ensure appropriate selectivity and sensitivity. Comparing the new RP-HPLC method with the previous ones, there is a noticeable difference in the achieved limits of detection and quantification. In the developed method, the detection limit for nicotine, cotinine and *trans*-3′-hydroxycotinin is 1.50 ng mL^−1^, 1.59 ng mL^−1^ and 1.47 ng mL^−1^, respectively. Using *HPLC-QQQ-MS/MS*, the LOD values were even more spectacular achieving the levels of 0.04, 0.02, 0.07 ng mL^−1^ respectively. Miller et al. [38] reported a similar detection limit of 1 ng/mL for all analytes for LC-MS/MS with electrospray ionization (ESI) using multiple reaction monitoring (MRM). Whereas McGuffey et al. [40] reported the detection limits of 1.94, 1.55, 3.53 ng/mL for *trans*-3′-hydroxycotinine, nicotine, cotinine respectively in LC-MS/MS measurements. Usually, the limit of quantification achieved by LC-MS/MS are smaller like 0.36, 0.32 ng/mL for nicotine, cotinine, respectively [45]. Zuccaro et al. [21] described the HPLC-DAD method that provided the limit of detection for nicotine, cotinine and *trans*-3′-hydroxycotinin of 10, 5, and 5 ng mL^−1^, respectively. The method used a time-consuming gradient elution, which allowed the separation of the analytes in more than 20 min. Another advantage of our method is the possibility of isocratic elution in no more than 15 min. While most authors using MS detection, despite its exceptional sensitivity, use the gradient elution mode [26,27,28,29,30,33,34,38,40,46,47]. Until now, only a few papers reported the usefulness of the isocratic elution mode [25,37,39].

The high convergence of the results (the relative error <10%) obtained with the new method and the reference one in the test carried out on a tobacco smoker, as well as the satisfactory validation parameters prove the success of the new method in confirming and quantifying the exposure to nicotine and its metabolites. The new method can be an alternative tool in applications that prefer fast, cheap, reliable methods using standard laboratory equipment.

## 4. Materials and Methods

### 4.1. Standards and Reagents

Standards of (−)-nicotine, (−)-cotinine, *trans*-3′-hydroxcotinine, caffeine were purchased from Sigma-Aldrich (St. Louis, MO, US). Deuterated (±)-nicotine-d4, and (±)-cotinine-d3 standards were purchased from Merck (Darmstadt, Germany). Sodium hexafluorophosphate (NaPF_6_), ammonium formate (HCOONH_4_) eluent additive for LC-MS, LiChropur^™^, ≥99.0%, formic acid (HCOOH) 98–100% for LC-MS LiChropur^™^ were obtained from Sigma-Aldrich. 85% m/m phosphoric acid (H_3_PO_4_), and sodium hydroxide (NaOH) were obtained from POCH (Gliwice, Poland). Plasma Control was obtained from Recipe chemicals (München, Germany). Acetonitrile (ACN, C_2_H_3_N) and methanol (MeOH, CH_3_OH) of HPLC reagent grade were obtained from Merck (Darmstadt, Germany). Water purified by ULTRAPURE Millipore Direct-Q 3UV-R (Merck, Darmstadt, Germany) of the resistivity 18.2 MΩ cm was used to prepare the aqueous solutions.

### 4.2. RP-HPLC-DAD Enriched with Chaotropic Salt

The HPLC analysis was carried out using Elite LaChrom HPLC Merck-Hitachi (Merck, Darmstadt, Germany), equipped with a DAD detector (L-2455), column thermostat Jetstream 2 Plus (100,375, Knauer). Chromatographic separation was conducted at 30 °C on a reversed-phase column Agilent 5 HC–C18(2) (250 × 4.6 mm I.D., 5 µm, 180 A, Agilent Technologies, Santa Clara, CA, US). The mobile phase composed of 10% (*v*/*v*) acetonitrile in phosphate buffer was pumped through the chromatographic system at a flow rate of 1.0 mL min^−1^. The buffer concentration was 20 mM, whereas chaotropic salt concentration was 30 mM in the whole mobile phase. The initial buffer solution (0.1 M) was prepared by dissolving 0.5 mL 85% (m/m) orthophosphoric acid in 80 mL water and adjusting to pH = 2.7 with saturated sodium hydroxide solution. The pH values were measured with CPC-105 Elmetron pH-meter (Zabrze, Poland). The mobile phases were filtered with a Nylon 66 membrane filter (Merck, Darmstadt, Germany) with pore size 0.45 μm. The injection volume was 20 µL corresponding to the volume of the Rheodyne injector loop. The diode array detector was operated at 260 nm.

#### 4.2.1. Preparation of Stocks and Working Standard Solutions

Individual stock solutions of nicotine and its metabolites were prepared in methanol and stored at −20 °C. The calibration curve standards of each analyte at seven concentration levels in the range of 10.00 to 1200.00 ng mL^−1^ (10, 50, 200, 400, 700, 900, 1200 ng L^−1^) were prepared by serial dilution of the 20 µg mL^−1^ stock solutions with the mobile phase. Working solutions were prepared also for quality control samples (QCs) by dilutions of stock solution to obtain a final concentration of 100, 500, 1000 ng L^−1^. 

#### 4.2.2. The Recovery Study

The calibration curves were constructed by plotting the standard peak area versus the concentrations spiked in the blank artificial plasma matrix. A total 0.01 mL of artificial plasma matrix, which was free from nicotine metabolites were spiked with 0.05 mL of the appropriate standard solution. Then, 140 µL acetonitrile was added to each mixture, vortex 60 s and centrifuged at 9000× *g* for 30 min. The supernatant of each aliquot of plasma was diluted to 1 mL with the mobile phase. A 20 µL of the extract was injected directly into the HPLC column. The statistical parameters of the curve were estimated using linear regression analysis. The LOD and LOQ were derived via signal-to-noise ratio (SN). The LOQ is 10 times the standard deviation (σ) of the blank whereas the LOD is related to 3σ. Recoveries and stability were examined using blank samples fortified with three different levels of 100, 500, and 1000 ng L^−1^. Samples of each level were prepared in six replicates. The relative standard deviation (RSD) values represent the variability and repeatability of the method.

#### 4.2.3. Preparation of the Plasma Samples from the Smoker

Human plasma samples were collected by venipuncture (approximately 10 mL of whole blood) from a healthy volunteer who gave informed consent, 15 min after smoking the cigarette. The sample was immediately centrifuged at 870 g for 10 min to obtain plasma. The plasma from the smoker was analyzed maintaining the fixed plasma to acetonitrile ratio (1:14). To 1.45 mL of plasma, 20 mL of acetonitrile was added, then vortex 10 min and centrifuged at 9000× *g* for 30 min. The obtained supernatant was lyophilized with freeze dryer Christ ALPHA 2-4 LD plus from Millrock Technology (Kingston, NY, USA), and the dry residue was dissolved in 300 µL of the mobile phase. The solution was divided into five vials, each with 50 µL. Then the samples were spiked with the same volume (50 µL) of the mixture containing increasing concentration of the analytes giving finally concentration of 10, 50, 100, 300, and 500 ng mL^−1^. The samples of 20 µL were injected directly into the HPLC column. The calibration curves of this new data set showed linear relationships between the analyte concentrations versus the peak areas measured at 260 nm of wavelength. The concentration of the analyte was determined by extrapolating the line to the x-axis of the calibration curve. The final results were calculated considering the dilution (2×) and concentrating steps (4.83×).

### 4.3. HPLC-QQQ-MS/MS 

#### 4.3.1. Apparatus and Detection Conditions

The LC-MS analysis was carried out using a triple quadrupole system (HPLC 1260 Agilent Technologies, Germany coupled to triple quadrupole mass spectrometer QqQ 6460, Agilent Technologies, USA) equipped with electron spray ionization (ESI) source. The chromatographic separations were performed using a ZORBAX RRHD StableBond C18 column 2.1 × 150 mm, 1.8 µm, (Agilent Technologies, USA). The column was heated to 50 °C. The analytes were eluted using a mobile phase consisting of 40% (*v*/*v*) acetonitrile in 5 mM ammonium formate (pH 4.5) in water pumped at a flow-rate of 300 μL min^−1^. The injection volume was 2 μL. The run time for HPLC-MS/MS method was 3.5 min. Mass spectrometric data were collected in positive ion mode, using multiple reaction monitoring mode. The ESI-MS/MS parameters and the retention time for the tested compounds are presented in Table 6.

#### 4.3.2. The Calibration Curve Preparation for the MS/MS Method

Calibration curve was prepared using plasma without nicotine and its metabolites. To 45 μL of clear plasma was added 5 μL of methanol solution of nicotine and its metabolites in proper concentration (to obtain levels 2, 5, 10, 20, 100 and 200 ng mL^−1^). Then was added 5 μL of deuterated internal standards (IS), i.e., nicotine-D4 and cotinine-D3 at a concentration of 100 ng mL^−1^ in MeOH. Samples were precipitated with 500 µL of frozen acetonitrile containing 0.1% HCOOH. The sample was centrifuged 2 min at 14,000 rpm, then the 500 µL supernatant was transferred to an Eppendorf vial (2 mL). It was evaporated to dryness under a stream of N_2_ at 40 °C (using a RapidVap^®^N_2_/48 rotary evaporator, Labconco, Kansas City, MO, USA). The dry residue was dissolved in 50 µL of the eluent and centrifuged for 2 min (at 14,000 rpm). The extracts were transferred to the inserts and analyzed with the HPLC-QQQ-MS/MS system.

#### 4.3.3. Method Validation

Quantitative analysis was performed using a calibration curve in the range of 2–200 ng mL^−1^. In order to validate the method, six replications were performed for each of the calculated parameters (repeatability and recovery) at two concentration levels. Determination of LODs (limits of detection) and LOQs (limit of quantification) was based on the calibration curve. The LOD values were calculated as 3 × σ/S where S is the slope of the calibration curve and σ is the standard deviation of the response, while the LOQ was calculated as 10 × σ/S.

#### 4.3.4. Sample Preparation

A total 50 μL of test plasma and 5 μL of deuterated internal standards (IS), i.e., nicotine-D4 and cotinine-D3 at a concentration of 100 ng mL^−1^ in MeOH, were added to a 2 mL test tube. Plasma with IS supplement was mixed and precipitated by adding 500 µL of frozen acetonitrile containing 0.1% HCOOH. The sample was centrifuged for 2 min at 14,000 rpm, then the 500 µL supernatant was transferred to an Eppendorf vial (2 mL). It was evaporated to dryness under a stream of N_2_ at 40 °C (using a RapidVap^®^N_2_/48 rotary evaporator). The dry residue was dissolved in 50 µL of the eluent and centrifuged for 2 min (at 14,000 rpm). The extracts were transferred to the inserts and analyzed with the HPLC-QQQ-MS/MS system. 

## 5. Conclusions

The paper compares two methods used for the determination of nicotine and its metabolites in human blood plasma, i.e., HPLC-QQQ-MS/MS and RP-HPLC-DAD enriched with the addition of chaotropic salt. Both methods met the validation criteria for the determination of xenobiotics in biological matrices regarding linearity, accuracy, repeatability, detection, and quantification limits. It should be emphasized that our work presents, for the first time, a reversed-phase HPLC chromatographic system enriched with chaotropic additives, for this purpose. RP-HPLC-DAD belongs to classic techniques, which are widely applied in analytical laboratories. We have proven that it can be easily adapted to study nicotine metabolites. Despite, the quantification limits of the HPLC-QQQ-MS/MS method being characterized by several times lower values, the developed chaotropic RP-HPLC-DAD method appears to be sufficient for toxicological and epidemiological studies of selecting patients exposed to tobacco smoke.

## Figures and Tables

**Table 1:** The statistical parameters of the calibration curves (y = ax + b).

Compound	Slope (a) ± s_a_	Intercept (b) ± s_b_	R^2^	se ^1^	F ^2^	LOD [ng ml^−1^]	LLOQ [ng ml^−1^]
*trans*-3′-hydroksycotinine	159.47 ± 3.54	2143.35 ± 1962.59	0.9980	2975.1	2024.92	1.47	4.42
cotinine	146.14 ± 2.46	1238.95 ± 1364.26	0.9989	2068.1	3519.14	1.59	4.78
nicotine	153.49 ± 3.44	2491.18 ± 1902.49	0.9980	2884.0	1996.34	1.50	4.51

^1^ The standard error of estimate (se); ^2^ Fisher F statistic.

**Table 2:** The recoveries of *trans*-3′-hydroxycotinine, cotinine, nicotine from the artificial plasma samples with intra- and inter-day precision.

Compound	Analyte Concentration [ng ml^−1^]	Intra Day Precision		Inter Day Precision	
		Extraction Yield [% ± SD]	Repeatability [CV]	Extraction Yield [% ± SD]	Repeatability [CV]
*trans*-3′-hydroxcotinine	100	96.02 ± 2.57	2.67	87.96 ± 3.06	3.47
	500	102.22 ± 0.53	0.52	104.61 ± 2.14	2.04
	1000	100.04 ± 2.35	2.35	101.02 ± 1.98	1.98
cotinine	100	93.49 ± 0.32	0.34	97.40 ± 1.63	1.67
	500	100.03 ± 1.60	1.59	102.93 ± 2.00	1.95
	1000	98.02 ± 2.71	2.76	100.37 ± 2.03	2.02
nicotine	100	100.27 ± 3.88	3.87	112.35 ± 6.31	5.61
	500	100.68 ± 2.34	2.32	100.60 ± 0.85	0.85
	1000	97.94 ± 2.71	2.77	94.66 ± 3.10	3.28

**Table 3:** The standard additions calibration method for the determination of nicotine and its major metabolites in plasma of tobacco smoker.

Compound	The Calibration Curve		The Plasma Sample [ng mL^−1^]		
	The Linear Equation	R^2^	Conc. in Sample	Conc. in Plasma	SD
Nicotine	y = 267.70 x + 81,194	0.9882	51.02	20.92	0.61
Cotinine	y = 132.58 x + 17,314.44	0.9999	130.62	53.55	1.48
*trans*-3′-hydroksycotinine	y = 191.96 x + 3427.33	0.9817	17.85	7.39	0.08

**Table 4:** The statistical parameters of the calibration curves (y = ax + b).

Compound	Slope (a)	Intercept (b)	R^2^	LOD [ng mL^−1^]	LOQ [ng mL^−1^]
*trans*-3′-hydroksycotinine	0.004926	−0.003564	0.9990	0.07	0.15
cotinine	0.082795	0.017080	0.9990	0.02	0.06
nicotine	0.035986	−0.061937	0.9989	0.04	0.10

**Table 5:** Recovery and precision of the investigated analytes quantification evaluated by HPLC-QQQ-MS/MS.

Analyte	Analyte Concentration [ng mL^−1^]	Recovery %	Intra Day Precision CV%	Inter Day Precision CV%	Smoker’s Plasma Sample	
					Conc. [ng mL^−1^]	SD
*trans*-3′-hydroxycotinine	20	81.9%	2.9%	4.6%	7.544	0.714
	200	96.4%	1.4%	5.6%		
cotinine	20	83.2%	1.3%	5.1%	50.180	0.551
	200	94.8%	1.3%	4.5%		
nicotine	20	76.8%	2.1%	5.7%	19.588	0.001
	200	93.1%	1.8%	6.6%		

**Table 6:** ESI-MS/MS parameters and retention time for selected compounds.

Compounds	Precursor Ion [m/z]	Product Ion [m/z]	Fragmentor [V]	Collision Energy [V]	Polarity	Retention Time [min.]
*trans*-3′-hydroxcotinine	193	134	144	20	Positive	1.77
		80		28		
cotinine	177	98	144	20	Positive	2.13
		80		28		
cotinine-d3	180	101	116	24	Positive	2.13
		80		28		
nicotine	163	130	116	20	Positive	1.79
		117		28		
nicotine-d4	167	136	116	16	Positive	1.80
		134		20		

## Data Availability

The data presented in this study are available upon request from Jolanta Flieger.
